# Cost-utility analysis of genomic profiling in early breast cancer in Colombia

**DOI:** 10.1186/s12962-023-00449-5

**Published:** 2023-07-10

**Authors:** Leonardo Rojas, María X. Rojas-Reyes, Diego Rosselli, Juan Guillermo Ariza, Alejandro Ruiz-Patiño, Andrés F. Cardona

**Affiliations:** 1 Thoracic and GU Unit, Fundación Centro de Tratamiento en Investigación Sobre Cáncer Luis Carlos Sarmiento Angulo (CTIC), Carrera 14 # 169 -49, Office 204, Bogotá, Colombia; 2 Molecular Oncology and Biology Systems Research Group (Fox-G), Universidad El Bosque, Bogotá, Colombia; 3 Institut d´Investigació Biomédica Sant Pau (IIB SANT PAU), Barcelona, Spain; 4 Department of Clinical Epidemiology and Biostatistics, Faculty of Medicine, Pontificia Universidad Javeriana, Bogotá, Colombia; 5 Head Medical Affairs, Baxter International, Bogotá, Colombia; 6 Foundation for Clinical and Applied Cancer Research-FICMAC, Bogotá, Colombia; 7 Direction of Research, Science and Education, Fundación Centro de Tratamiento en Investigación Sobre Cáncer Luis Carlos Sarmiento Angulo (CTIC), Bogotá, Colombia

**Keywords:** Breast cancer, Personalized medicine, Cost-utility

## Abstract

**Background:**

In Colombia, the best strategy to establish indication for adjuvant chemotherapy in early breast cancer (EBC) remains unknown. This study aimed to identify the cost-utility of Oncotype DX™ (ODX) or Mammaprint™ (MMP) tests to establish the necessity of adjuvant chemotherapy.

**Methods:**

This study used an adapted decision-analytic model to compare cost and outcomes of care between ODX or MMP tests and routine care without ODX or MMP tests (adjuvant chemotherapy for all patients) over a 5-year time horizon from the perspective of the Colombian National Health System (NHS; payer). Inputs were obtained from national unit cost tariffs, published literature, and clinical trial database. The study population comprised women with hormone-receptor-positive (HR +), HER2-negative, lymph-node-negative (LN0) EBC with high-risk clinical criteria for recurrence. The outcome measures were discounted incremental cost-utility ratio (ICUR; 2021 United States dollar per quality-adjusted life-year [QALY] gained) and net monetary benefit (NMB). Probabilistic (PSA) and deterministic sensitivity analysis (DSA) were performed.

**Results:**

ODX increases QALYs by 0.05 and MMP by 0.03 with savings of $2374 and $554 compared with the standard strategy, respectively, and were cost-saving in cost-utility plane. NMB for ODX was $2203 and for MMP was $416. Both tests dominate the standard strategy. Sensitivity analysis revealed that with a threshold of 1 gross domestic product per capita, ODX will be cost-effective in 95.5% of the cases compared with 70.2% cases involving MMP.DSA showed that the variable with significant influence was the monthly cost of adjuvant chemotherapy. PSA revealed that ODX was a consistently superior strategy.

**Conclusions:**

Genomic profiling using ODX or MMP tests to define the need of adjuvant chemotherapy treatment in patients with HR + and HER2 −EBC is a cost-effective strategy that allows Colombian NHS to maintain budget.

**Supplementary Information:**

The online version contains supplementary material available at 10.1186/s12962-023-00449-5.

## Background

Breast cancer tumors are the most frequently occurring type of tumors; this cancer type is the leading cause of cancer-related death in women in Colombia, with annual incidence of 48.3 and mortality of 13.1 per 100,000 women [1]. Adjuvant chemotherapy reduces the risk of recurrence and increases overall survival; however, it involves a risk of associated toxicity, decreased quality of life, and a significant burden on the healthcare system [2–4]. The risk of recurrence determines a patient’s eligibility for adjuvant chemotherapy. Several clinicopathological parameters are considered to determine the risk of recurrence, such as lymph node involvement, tumor size, subtype and histological grade, lymphovascular invasion, proliferation markers, hormone-receptor (HR) status, and HER2/neu [5, 6]. Adjuvant chemotherapy may not be recommended in patients with HR-positive ( +) and HER2-negative (−) early breast cancer (EBC) [2]. In this population, other clinical criteria, such as tumor size, the degree of differentiation, and patient age, can be taken into consideration [7]. However, recommendation based solely on clinical criteria may lead to a significant proportion of these patients being exposed to the adverse effects of chemotherapy and deterioration of quality of life with limited or uncertain benefit [8].

In the last decade, quantitative tests evaluating gene expression using microarray and reverse-transcription polymerase chain reaction techniques were developed to better establish the prognosis of patients with EBC with an HR + and HER2 −status. Two of these techniques are Oncotype DX™ (ODX; Genomic Health, Redwood City, CA) and Mammaprint™ (MMP; Agendia, Irvine, CA) [9, 10]. Both techniques have been validated in different studies and proven more accurate at estimating the recurrence risk compared with clinical parameters and other algorithms such as Adjuvant! Online and Nottingham Prognostic Index (NPI) [11–13]. Furthermore, there are no relevant variations in test results among genetically different populations such as Latin-American patients [14].ODX and MMP are recommended by different breast cancer treatment guidelines in the word [15, 16]. In Colombia, according to guidelines recommendations, and clinical practice, the decision about adjuvant chemotherapy in patients with HR + and Her2 −EBC is based on clinical and pathologic characteristics, and Oncotype DX™ (ODX) or Mammaprint™ (MMP) are recommended and approved for the support this decision [17]. Despite the costs of performing ODX or MMP assumed by the Colombian National Health System (NHS), the payers demand economic evidence to understand the trade-offs associated with funding those technologies.

Direct costs of care for patients with breast cancer are high, and a significant proportion of these are incurred from chemotherapy, which includes treatment of its side effects [18]. Therefore, a strategy that allows an adequate estimate of the risk of recurrence and identification of patients who will benefit from adjuvant chemotherapy will facilitate healthcare cost reductions. In an emerging country like Colombia, with limited economic resources, finding strategies that provide a personalized care and cost savings are strongly needed. This study aimed to determine which of the following interventions was more cost-effective for patients with HR + and HER2 −EBC with high-risk clinical criteria defined as that used in the Microarray in Node-Negative Disease May Avoid Chemotherapy trial: [19] administer adjuvant chemotherapy in all patients (standard intervention) or define treatment indication based on results from ODX or MMP genomic tests.

Our results could help to solve these questions in other countries with emerging economies. Health economics evaluations would help define the recommendations for managing diseases and funding decisions. Our study is the first economic study that evaluates this topic in Colombia and also the first economic study that evaluates ODX and MMP among Hispanics.

## Methods

### Overview

An economic cost-utility study evaluating the performance of ODX or MMP test was performed in a cohort of patients with HR + and HER-2 − EBC, without lymph node involvement (LN0) and with high risk of recurrence [tumor size (T) of > 3 cm or > 2 cm and a degree of differentiation of 2 or a T of > 1 cm, and a degree of differentiation of 3] according with the criteria used in MINDACT trial [19]. (Table 1 summarizes the scope of our model). Following the suggestions of the local health technology assessment agency for economic evaluations in health-Instituto de Evaluación Tecnológica en Salud-(IETS), we consider a cost-utility threshold according to gross domestic product (GDP) per capita [20, 21]. The GDP per capita data were obtained from the *Banco de la República* reports for the year the evaluation was performed (2021; 1 GDP = 3281 USD) [20]. Technologies with ICUR below 3 GDP are considered cost-utility according to local agency recommendations (IETS) [22]. ICUR (2021 United Sates dollar [USD] per QALY gained) and net monetary benefit (NMB) were estimated. The discount rate used was 5% per annum according to local agency recomendations [22].

**Table 1:** Scope of the economic analysis

Element economic analysis	Description
Population	Women with HR + and HER2– early-stage breast cancer (LN0) with high-risk clinical criteria as per the MINDACT trial [19]
Interventions	1→Oncotype DX (cutoff points as per the TAILORx trial) [23] 2→Mammaprint (cutoff points as per the MINDACT trial) [19]
Comparator	Chemotherapy for all
Primary health economic outcome	Incremental cost per QALY gained and net monetary benefit
Perspective	Colombian NHS
Time horizon	5 years
Discount rate	5% per annum. A sensitivity analysis was performed with discount rates of 0%, 3.5%, and 7% [24]
Price year	2021

*NHS* National Health System, *HR* +  HR-positive, *HER2* −  HER2-negative, *LN0* without lymph node involvement, *QALY *quality-adjusted life-years

The model was developed using Microsoft Excel™. This was considered a risk-free study according to local laws (Resolution 8430 of 1993) [25] and was approved by the research and ethics committee of the Faculty of Medicine of the Pontificia Universidad Javeriana, Bogotá, Colombia (Ref. 2018/41).

### Utility

Quality-adjusted life-years (QALYs) were considered as a measurement of care outcomes. Following the suggestion of IETS in the absence of local information, utilities were taken from the literature [22]. Utility valuation weights used were those calculated for the Latin-American population in the USA in 2008 [26]. In this study, a national population survey was conducted in the United States in 2002, based on a sample of 1603 non-Hispanic nonblacks and 1115 Hispanics. Participants provided time trade-off utilities for a subset of 42 EQ-5D health states. The utilities for each health state of the model: recurrence and free of recurrence, and these ranges were extracted from the literature [27–29]. The utility of breast cancer adjuvant chemotherapy was taken from Tengs TO et al. study [27]. The discount rate was 5% per annum. The values of utilities used are shown later in Table 2.

**Table 2:** Variables, values, and parameters used for DSA and PSA

Variable	Base case	Range DSA		Distribution and parameters PSA			References
		Lower	Higher	Distribution	⍺	β	
Chemotherapy							
DRFS 5 years	0.939	0.925	0.950	Log-normal	− 0.06	0.01	Database NCT00310180
OS 5 years	0.966	0.955	0.974	Log-normal	− 0.03	0.01	
Chemotherapy							
DRFS 5 years	0.939	0.925	0.950	Log-normal	− 0.06	0.01	Database NCT00310180
OS 5 years	0.966	0.955	0.974	Log-normal	− 0.03	0.01	
ODX high risk							
DRFS 5 years	0.914	0.895	0.930	Log-normal	− 0.09	0.01	Database NCT00310180
OS 5 years	0.957	0.943	0.968	Log-normal	− 0.04	0.01	
ODX low risk							
DRFS 5 years	0.972	0.963	0.979	Log-normal	− 0.03	0.00	
OS 5 years	0.974	0.965	0.981	Log-normal	− 0.03	0.00	Database NCT00310180
ODX high risk							
DRFS 5 years	0.914	0.895	0.930	Log-normal	− 0.09	0.01	
OS 5 years	0.957	0.943	0.968	Log-normal	− 0.04	0.01	Database NCT00310180
ODX low risk							
DRFS 5 years	0.972	0.963	0.979	Log-normal	− 0.03	0.00	
OS 5 years	0.974	0.965	0.981	Log-normal	− 0.03	0.00	
MMP high risk							
DRFS 5 years	0.909	0.880	0.932	Log-normal	− 0.10	0.01	[19]
OS 5 years	0.955	0.934	0.969	Log-normal	− 0.05	0.01	
MMP low risk							
DRFS 5 years	0.949	0.928	0.963	Log-normal	− 0.05	0.01	[19]
OS 5 years	0.970	0.953	0.981	Log-normal	− 0.03	0.01	
Cost							
ODX	$3551	$2841	$4261	Gamma	96.4	362.36	Provider
MPT	$3551	$2841	$4261	Gamma	96.4	362.36	Provider
Rem.1^st^ year^a^	$109.4	$76	$150	Gamma	33.84	18.80	[31], [32]
Rem. ≥ 2^nd^ year^a^	$53.21	$32	$83	Gamma	16.58	13.07	[31], [32]
Adjuvant chemotherapy^a^	$464.38	$243	$807	Gamma	10.44	148.01	[31], [32]
Recurrence^a^							
Palliative care^a^	$4587.45	$4248	$4935	Gamma	684.17	180.64	[31], [32]
Adverse event^a^	$248.95	$213	$300	Gamma	125.06	22.93	[31], [32]
Utility^a^							
Recurrence-free survival	0.9	0.85	0.95	Beta	7.50	92.50	[27, 28]
Recurrence							
Adjuvant chemotherapy	0.55	0.44	0.66	Beta	2.42	97.58	[27–29]
	0.74	0.59	0.89	Beta	6.17	93.83	[27]
Adverse event	0.07	0.02	0.08	-	–	–	[35], [36]

^a^ monthly

*DSA* deterministic sensitivity analysis, *PSA* probabilistic sensitivity analysis, *⍺ y β* probabilistic distribution parameters, *DRFS* distant recurrence-free survival *OS* overall survival, *ODX* Oncotype DX, *MMP* Mammaprint, *Rem* remission

### Resource use and cost

The costs were reported as the value of 2021 USD (1 USD = 3850 Colombian pesos, according to an average of 2021 COP market exchange rate obtained from Banco de la República reports) [30], and only direct costs were accounted for.

The following sources were used to estimate costs: [1] In MMP and ODX genomic tests, the prices in the Colombian market consulted with the providers (the cost of both tests was regulated by market laws and local regulatory agency) were used and (2) cost-generating events and frequency of use for each state were identified through (a) clinical recommendations from clinical practice guidelines and (b) a panel of experts. Costs were taken from local tariff manuals, estimated for 2019, and adjusted for yearly inflation for 2021 [31–33]. Twelve oncologists and breast surgeons conformed to the panel of experts. They were asked about cost-generating events, healthcare resources and frequency of use. None declared conflicts of interest. The disagreement was resolved by consensus. Additional file 1: Table S1 in summarizes the resources, cost, and references used.

In this study, only febrile neutropenia as adverse event of chemotherapy was taken into account considering that it is the most frequent severe adverse event of adjuvant chemotherapy in breast cancer [34–36]. The chemotherapy regimen considered for this trial was anthracycline and taxane regimen (doxorubicin 600 mg/m^2^/cyclophosphamide 600 mg/^2^ × 4 cycles q/21 days → Paclitaxel 80 mg/m^2^ × 12 weeks) which is the most frequent regimen using according with practice guidelines and expert consensus.

A type or base case was designed. This base case involved the use of cost-generating events (i.e., consultations, medications, diagnostic tests, hospitalization, and procedures). Subsequently, the monetary cost for the Colombian NHS of each of the cost-generating events was included.

### Model description

The model structure was developed by consensus among the authors, and it took into account previous published and validated models [37]. It consisted of an initial decision tree followed by a three health-state time-dependent discrete-state transition (Markov) cohort model with one-month cycles for survival. The model followed the clinical pathway that is in accordance with the recommendations of the clinical practice guidelines under the assumption that it corresponds to the best available description of usual clinical practice in Colombia [17].

An analytical decision model was developed in which a cohort of patients with HR + , HER2 − , and LN0 EBC with high-risk clinical criteria was assigned to the standard intervention in which all patients received chemotherapy or underwent ODX or MMP and, according to the results, were classified as high or low risk.

For patients assigned to the standard intervention (chemotherapy for all), distant recurrence-free survival (DRFS) and overall survival (OS) were estimated according to the information available from the TAILORx trial database (NCT00310180).

From this database, we selected the population that met the high-risk clinical criteria, i.e., a tumor size (T) of > 3 cm or > 2 cm and a degree of differentiation of 2 or a T of > 1 cm, and a degree of differentiation of 3, according with the criteria used in MINDACT trial [19]. DRFS and OS were estimated for patients who met these criteria and subsequently underwent chemotherapy.

**Fig. 1:**
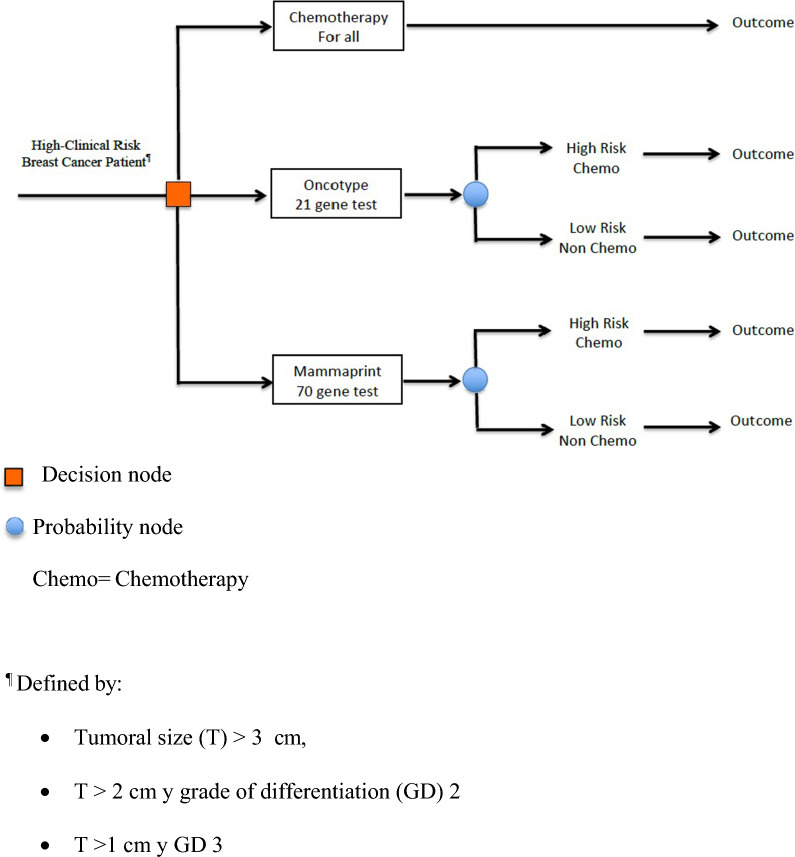
Recurrence risk classification algorithm

**Fig. 2:**
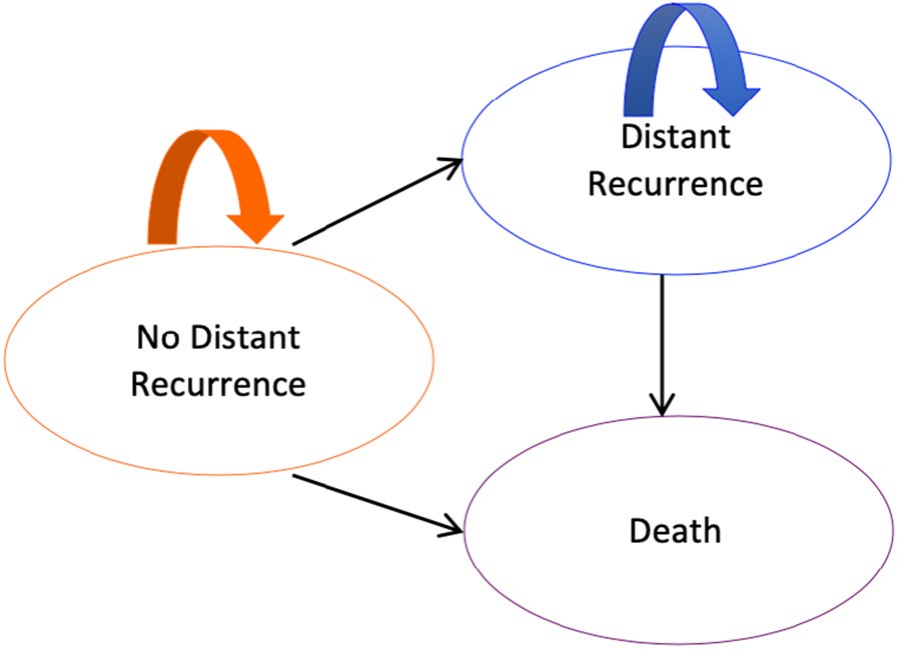
Markov Model

In ODX, this same database was considered, and patients were categorized as high or low risk according to the definitions of the TAILORx trial [23], where high risk corresponds to a score of ≥ 16 for patients aged ≤ 50 years or a score of ≥ 26 for those aged > 50 years and low risk corresponds to a score of ≤ 15 for patients aged ≤ 50 years and ≤ 25 for those aged > 50 years. DRFS and OS were similarly estimated. In MMP, the possibility of a high- or low-risk classification was considered, and the risk of recurrence and death for each risk category was based on the report of the MINDACT trial in a population with negative nodes (N0) [19]. For both tests, high-risk patients received adjuvant chemotherapy, and low-risk patients did not (Fig. 1). The cohort was modeled using a Markov process that had the following three mutually exclusive health states: distant recurrence (meaning metastatic breast cancer), distant recurrence-free, and death (death from other causes or breast cancer) (Fig. 2). Initially, all patients were in a recurrence-free state and could subsequently progress into a state of recurrence before dying from breast cancer. Patients who did not present with cancer recurrence had a constant probability of dying from other causes. The events of interest were modeled according to the transition of patients from one state to another in 1-month cycles. Their effect on QALY was estimated according to literature reports for these states [27–29]. Similarly, the impact on QALY in patients undergoing chemotherapy was based on literature reports [27].

### Model assumptions

The most important assumption in our model is the estimation made for QALYs and utilities; however, the process was done according to the recommendations for the country in this regard [22]. By contrast, for the population receiving the standard intervention (chemotherapy), the estimates were made from the database of a clinical study that might not reflect the conditions of routine clinical practice, and there may be differences according to patient race. However, both clinical characteristics and treatments that these patients received were similar to those recommended in different clinical practice guidelines and were contrasted with the panel of experts [17]. Furthermore, the database used for modeling contained Latin population data. It was assumed that there was 100% adherence to the result of ODX or MMP—if the result corresponded to a high-risk status, the patient was assumed to require chemotherapy, and if low risk, no chemotherapy was needed. However, this trend has been reported in a lower percentage in a previous case series [38].

By the time of the first analysis, overall survival, and disease-free survival results of one of the trials included in our model had been reported to five years of follow-up [19]. For this reason, our model's temporal horizon was five years to avoid survival assumptions.

### Statistical analysis

Deterministic sensitivity analysis (DSA) and probabilistic sensitivity analysis (PSA) were performed. A univariate DSA was developed for the assumptions, probabilities, utilities, and costs, whereas each input was varied in turn, with all other parameters fixed. The ranges used in the DSA were based on the confidence intervals of the studies and on extreme values taken from the literature and sources consulted (e.g., expert panel). A tornado diagram was used to show the effect of the variation in these parameters on incremental NMB. To evaluate the overall uncertainty of any parameter, PSA was performed using the Monte Carlo simulation method by randomly sampling from distributions assigned to model input parameters according to the nature or behavior of the variable. The simulation was applied 1,000 times to ensure model reproducibility. The result is shown in a scatter plot. Table 2 enlists the variables used as inputs for our model and their values, ranges, and distribution parameters applied in DSA and PSA.

## Results

### Clinical parameters of patients with high-risk status

Among the 10,273 patients included in the NCT00310180 study, information was available to establish the high-risk criteria for 10,086 patients (98.17%). In this population, 2981 patients (20.35%) met the high-risk clinical criteria, the median age was 56.3 years (95% confidence interval 56.0–56.7 years), and 68.6% were postmenopausal. The probability rates of high and low genomic risks with ODX test in high and low clinical risk populations were 42.1% and 57.9%, respectively. Furthermore, 75% of patients aged ≤ 50 years and 28.8% of patients aged > 50 years presented a high genomic risk. The population of high clinical risk that was randomized to receive chemotherapy corresponding to the standard intervention of our analysis was 1643, which corresponded to 55.1% of the total high–clinical risk population. Additional file 1: Table S2 in summarizes the main characteristics of the population selected from NCT00310180 study for our analysis.

### Survival analysis

Among the patients who met the high-risk clinical criteria in the ODX group (selected from NCT00310180 study database), the estimated 5-year DRFS was 91.4% and 97.2% for patients with high and low genomic risks, respectively. The estimated OS rates for this population were 95.7% and 97.4% for patients with high and low genomic risks, respectively.

In the MMP group, the 5-year DRFS rates were 90.9% and 94.9% for patients with high and low genomic risks, respectively. The OS rates for those with high and low genomic risks were 95.5% and 97%, respectively, according to the report of the MINDACT trial for patients with LN0 disease [19].

For the population who underwent chemotherapy (standard intervention), the estimated 5-year DRFS rate was 94%, and the OS rate was 97%. Additional file 1: Figure S1A and S1B in Additional file 1 shows the 5-year DRFS and OS for ODX, MMP, and standard intervention.

### Base case analysis

According to the base case analysis for a 5-year time horizon, the total cost for patients who received the standard intervention (chemotherapy for all) was $13,445.49, and the QALY value for this group was 3.76. For the standard intervention, ODX had a higher QALY (3.82) than MMP (3.79). The average cost for the 5-year time horizon for ODX and MMP groups was lower than that of the standard intervention, i.e., $11,071 and $12,892, respectively, which translated into savings of $2750 for ODX and $755 for MMP. ODX and MMP strategies were dominant in the cost-effective plane (Additional file 1: Figure S2).

In addition, the two genotyping strategies represented an incremental NMB of $2203 for ODX and $416 for MMP. This was considered when a willingness to pay (WTP) threshold of 1 GDP per capita was considered the base. When two ODX tests were compared (a simple indirect comparison), ODX was more cost-effective than MMP. Table 3 summarizes the findings above.

**Table 3:** Cost-utility results

	Chemotherapy (ChT)	Oncotype DX™ (ODX)	Mammaprint™ (MMP)
LY	4.36	4.33	4.34
QALY	3.75	3.81	3.79
Costs	$13,446	$11,071	$12,892
Incremental LY			
Test vs. ChT		− 0.03	− 0.02
ODX vs. MMP		− 0.01	
Incremental QALY			
Test vs. ChT		0.06	0.03
ODX vs. MMP		0.03	
Incremental cost			
Test vs. QT		− $2375	− $554
ODX vs. MMP		− $1821	
ICUR (per QALY)			
Test vs. QT		Dominant	Dominant
ODX vs. MMP		Dominant	
Incremental NMB (per QALY)			
Test vs. ChT		$2751	$755
ODX vs. MMP		$1996	

*LY* life-year, *QALY* quality-adjusted life-year, *ICUR* incremental cost-utility ratio, *NMB* net monetary benefit

### Sensitivity analysis

Regarding DSA, variables that were considered at the discretion of the researchers and the panel of experts were defined as having greater uncertainty for the model, which included (a) utilities during adjuvant chemotherapy, recurrence, and drug-free period recurrence and (b) costs for chemotherapy, adverse events, treatment to recurrence, and palliative care. Additional file 1: Fig. S3 in shows the tornado diagram for the DSA for ODX in QALY.

In our analysis, the variable with the greatest influence was the monthly cost of adjuvant chemotherapy. Notably, we observed that the utility values variation did not significantly affect. This suggested that despite the limitations imposed by their estimation from populations other than that of Colombia, they do not significantly affect our findings.

**Fig. 3:**
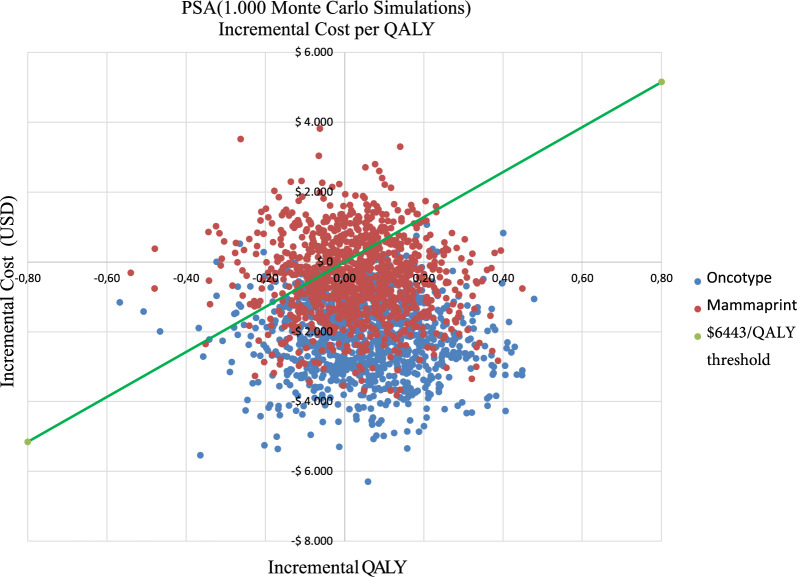
Probabilistic senstivity analysis

Similar findings were obtained in the DSA for MMP (Additional file 1: Fig. S4). Given that chemotherapy treatment costs < $274, MMP may not be cost-effective. In the PSA, considering that a threshold of 1 GDP per capita for 2021 corresponded to $6443, ODX had 95.5% probability of being cost-effective, and MMP had 70.2% compared with the standard strategy (chemotherapy for all patients). Figure 3 shows the PSA for QALY.

## Discussion

The use of adjuvant chemotherapy in EBC has been beneficial to some patients by decreasing tumor recurrence and increasing OS; however, in some instances, chemotherapy can generate serious adverse events that cause deterioration in the quality of life and a substantial increase in health costs [2, 4, 39]. Furthermore, in this population, it is typically difficult to determine whether chemotherapy should be administered [40].

Gene expression profiles such as MMP and ODX have established more precisely the prognosis and helped define the benefit of chemotherapy treatment in an individual assessment. This results in the accurate selection of patients and avoids unnecessary therapies; however, performing these tests may imply an additional cost [19, 23]. These tests are recommended by different clinical practice guidelines, especially for patients with a high clinical risk, and they are often used in Colombian oncology practice [17].

In this study, possibly the first in Colombia, we found that performing ODX or MMP is a cost-utility strategy for the health system and generates economic savings. In our model, although we observed an increase in life-years after comparing the performance of standard strategy (4.36) with that of MMP (4.34) or ODX (4.33), these results are at the expense of deterioration in the quality of life that can be attributed to chemotherapy treatment, resulting in a benefit in terms of QALY that favors MMP or ODX (0.03 and 0.05, respectively). Despite the possibility of these differences in improvements in both life-year and QALY not being statistically or clinically significant, the capability of MMP or ODX tests to better select patients for chemotherapy results in reducing the cost of chemotherapy treatment. The NMB for ODX is $2203, and that for MMP is $416, indicating that genomic profiling using these tests generates an economic surplus compared with the standard strategy in the WTP threshold defined for Colombia as 1 GDP per capita for the QALY analysis. In this case, the cost to obtain the benefit is less than the maximum amount that the Colombian NHS would consider paying for this benefit. In the sensitivity analysis, MMP could not be considered economically acceptable only when the costs of adjuvant chemotherapy were < $274 per month of treatment.

Notably, even when the system is unwilling to pay any cost for this benefit, i.e., with a WTP threshold of $0, both tests are cost-effective (incremental NMB for ODX at $2203 and incremental NMB for MMP at $416).

From the perspective of the Colombian NHS, for a WTP of 1 GDP per capita, there is a 95.5% and 70.2% probability that ODX and MMP tests will be cost-effective, respectively. Importantly, even with a WPT of COP 0, the probability that the tests are cost-effective is high, especially for one of the tests (99.1% for ODX and 66.7% for MMP).

Our results are in agreement with those reported previously in the literature. A cost-utility study of the genomic profile for breast cancer conducted in Canada that included information from the TAILORx [23] and MINDACT [19] trials showed that the genomic profiling of breast cancer patients using ODX or MMP tests is a cost-effective strategy below the threshold of WTP defined for this study when compared with the standard management, i.e., the absence of any test. In this analysis, ODX has an 89.2% probability, and MMP has an 89.2% probability of being cost-effective for a WTP threshold of Canadian dollar 50,000 [41].

Several systematic reviews have concluded that performing the genomic profile in EBC to define adjuvant chemotherapy treatment is a cost-effective strategy [42–44]. However, other analyses have shown that this finding is inconsistent in all population subgroups and that ODX genomic profile is cost-utility when performed in a high–clinical risk population and not in a low–clinical risk population. A cost-utility study conducted by the UK National Institute for Excellence in Health and Care found that neither ODX nor MMP was cost-utility from the perspective of the UK health system [45]. No predictive role of the benefit of chemotherapy was considered for ODX or MMP. Notably, at the time of performing this analysis, the results of the TAILORx trial were unknown, which demonstrated the ability of ODX to establish not only prognosis but also the benefit of adjuvant chemotherapy [23]. When the predictive role of ODX to establish the benefit of adjuvant chemotherapy was included, this test was cost-utility, particularly for patients with high clinical risk (for this study defined as the subgroup with NPI > 3.4), a finding similar of ours [45]. In MMP, despite the results of the MINDACT trial [19], this test was not cost-utility [45].

Several cost-utility studies have not considered this analysis for clinical risk subgroups, which could favor the new test as cost-utility, and for this reason, the incorporation of clinical characteristics into the cost-utility models is recommended [46]. In contrast, the performance of MMP is only recommended in patients with high clinical risk, and combining these clinical criteria with ODX results can increase its prognostic capacity [47, 48]. Only patients with high-risk clinical criteria were included in our model, which is a conservative strategy, demonstrating that performing MMP or ODX in this population is a cost-utility strategy. Hall et al. found results similar to ours in the United Kingdom when they used a model that also included patients with a high clinical risk with lymph node involvement [37]. In addition to MMP and ODX, other tests such as PAM-50 (Prosigna™), MammaTyper™, IHC4, and IHC4-AQUA™ (NexCourseBreast™) were also evaluated by Hall et al. and found an 86% probability that gene expression profiles are cost-utility in defining the need for adjuvant chemotherapy in patients with EBC [37].

In our study, when two tests were compared, ODX was more cost-effective than MMP, with an incremental NMB of $1787 and a 99% probability of being more cost-effective, however, this indirect comparison should be taken with caution; a head-to-head comparison will be needed to show differences between tests. Our findings suggest that to achieve results similar to the ODX test, the costs of the MMP test should be lower.

Most cost-utility studies have assumed that the relative risk reduction (RRR) for distant recurrence attributed to chemotherapy varies according to different genomic risk groups, i.e., the RRR is 0 for patients with low genomic risk and higher for those with high genomic risk. These assumptions make genomic testing more cost-utility because it can better establish the magnitude of chemotherapy benefit than the traditional clinical criteria. However, the predictive values of these tests for these cost-utility studies were based on limited information based on retrospective analysis [49, 50]. In our model, we considered data from prospective studies with a significant number of patients in which the predictive role of the tests has been demonstrated, particularly for ODX [19, 23].

Our study has limitations. Foremost, we were not able to identify QALY for our population. Despite being a methodological limitation, some guidelines recommend QALY as an outcome measure because this measure more comprehensively evaluates health outcomes [22]. This limitation was accepted by the local economic evaluation agency (IETS) considering the absence of QALY data for Colombia. For our case, it is essential to establish the effect of chemotherapy on the quality of life, and according to our results, they are significant when evaluated from this perspective. Despite these limitations, variations that could exist in the valuations of the utilities do not alter our results, as evidenced by the DSA. Although there could be utility variations among different populations, regardless of their significance, these variations did not seem to affect our results. However, it is essential to assess QALYs and the utilities for the Colombian population so that more accurate cost-utility evaluations can be performed in different scenarios, especially in those closely related to the quality of life, such as oncological diseases. As shown in our analysis, if measures of effectiveness, such as years of life, are analyzed, a treatment or strategy will be considered not cost-effective, as its effect on the quality of life will be ignored. Another limitation of our study is the 5-year time horizon, which is relatively short compared to other cost-utility studies. This time horizon includes the main relevant outcomes, especially the secondary event associated with chemotherapy considered in our model, which was febrile neutropenia. In this regard, our model is conservative, as it does not account for other adverse events attributable to chemotherapy, such as heart failure or the development of secondary malignancies. These adverse events have a negative effect on the quality of life, risk of death, and increased health costs. Furthermore, by the time of the analysis, only 5-year follow-up data were available for one of the trials used [19], and we preferred not to include survival assumptions.Besides, our model’s relatively short time horizon was enough to demonstrate differences.

Considering that genomic profiling using ODX and MMP tests is a cost-utility and cost-saving strategy, establishing the budget effect of these tests could be essential to define whether they can be included in Colombia’s health benefit plan.

This is the first economic study to the authors´ knowledge that evaluates both tests in Latam and was based on the data of the most important prospective trials of ODX and MMP. Our results could be generalizable to emerging economies.

## Conclusions

Genomic profiling using ODX and MMP tests is a cost-effective strategy for the Colombian NHS. This strategy generates savings in the health system compared to the standard treatment strategy. These tests should be indicated in a population with HR + , HER2 − EBC with high-risk clinical criteria.

## Supplementary Information

**Additional file 1: Figure S1.** Distant recurrence-free survival. **Figure S2.** Cost-utility plot. **Figure S3.** Deterministic sensitivity analysis of Oncotype DX. **Figure S4.** Deterministic sensitivity analysis of Mammaprint. **Table S1.** Patient characteristics. **Table S2.** Resources and costs.

## Data Availability

This manuscript was prepared using data from datasets [NCT00310180] from the National Clinical Trials Network (NCTN) Data Archive of the National Cancer Institute [51]. Data were originally collected from the clinical trial NCT00310180 (Adjuvant chemotherapy guided by a 21-gene expression assay in breast cancer). All analyses and conclusions in this manuscript are the sole responsibility of the authors and do not necessarily reflect the opinions or views of the clinical trial investigators, NCTN, or NCI.

## Abbreviations

DRFS: Distant recurrence-free survival
EBC: Early breast cancer
GDP: Gross domestic product
HR: Hormone-receptor
ICUR: Incremental cost-utility ratio
MMP: Mammaprint™
NCTN: National Clinical Trials Network
NHS: National Health System
NMB: Net monetary benefit
NPI: Nottingham Prognostic Index
OS: Overall survival
ODX: Oncotype DX™
PSA: Probabilistic sensitivity analyses
QALY: Quality-adjusted life-years
RRR: Relative risk reduction
USD: United States dollar
WTP: Willingness to pay
