# Supplementary material for: Cost-utility analysis of genomic profiling in early breast cancer in Colombia
Source: Cost Eff Resour Alloc. 2023 Jul 10;21:42. doi: 10.1186/s12962-023-00449-5 (PMC10334609; doi:10.1186/s12962-023-00449-5)
Supplement: Supplementary file 1 — Additional file 1: Figure S1. Distant recurrence-free survival. Figure S2. Cost-utility plot. Figure S3. Deterministic sensitivity analysis of Oncotype DX. Figure S4. Deterministic sensitivity analysis of Mammaprint. Table S1. Patient characteristics. Table S2. Resources and costs. [file 12962_2023_449_MOESM1_ESM.docx]

**Additional file Figure S1A: Distant recurrence-free survival.**

DRFS, distant recurrence-free survival; HR, high risk; LR, low risk; CT, chemotherapy

Distant recurrence free survival expected for each strategy.

**Additional file Figure S1B: Overall survival.**

OS, Overall survival; HR, high risk; LR, low risk; CT, chemotherapy

Overall survival expected for each strategy.

**Additional file Figure S2: Cost-utility plot**

QALY: Quality-adjusted Life-Year

Scatterplot on the incremental cost-utility plane, comparing each test with chemotherapy for all in the base-case analysis.

**Additional file Figure S3: Deterministic sensitivity analysis of Oncotype DX**

Cht, chemotherapy; m, month; AE, adverse event; NMB, net monetary benefit; Max, maximum; Min, minimum

One-way sensitivity analysis results of Oncotype

**Additional file Figure S4: Deterministic sensitivity analysis of Mammaprint.**

Cht, chemotherapy; m, month; AE, adverse event; NMB, net monetary benefit; Max, maximum; Min, minimum.

One-way sensitivity analysis results of Mammaprint

**Additional file Table S1: : Patient characteristics**

| **Characteristics** | **High clinical risk**  **N = 2981** | **High clinical risk with chemotherapy N = 1643** |
| --- | --- | --- |
| Age (median), n (%) | 56.3 y | 55.6 y |
| ≤ 50 y | 860 (28.9) | 511 (31.1) |
| > 50 y | 2,121 (71.2) | 1,132 (68.9) |
| Menopausal status, n (%) |  |  |
| Pre/Perimenopausal | 976 (31. 4) | 547 (33.3) |
| Postmenopausal | 2,048 (68.6) | 1,096 (66.7) |
| Oncotype risk, n (%) |  |  |
| High risk | 1,255 (42.1) | 1,013 (61.6) |
| ≤ 50 y and score ≥ 16 | 645 (21.6) | 447 (27.2) |
| > 50 y and score ≥ 26 | 610 (20.5) | 566 (34.4) |
| Low risk | 1,726 (57.9) | 630 (38.4) |

Clinical characteristics and genomic profile risk results (high or low) of population selected from NCT00310180 trial database

**Additional file Table S2: Resources and costs.**

|  | **Base-case** | **Sensitivity range** | | **Reference** |
| --- | --- | --- | --- | --- |
|  |  | **Minimum** | **Maximum** |  |
| Oncotype DX^TM^ | $ 3,551 | $2,841 | $4,261 | Provider |
| Mammaprint^TM^ | $ 3,551 | $2,841 | $4,261 | Provider |
| ChT regimen 1* | $308 | $161 | $534 | SISMED^32^,  SOAT 2019^33^ |
| ChT regimen 2** | $621 | $531 | $749 | SISMED^32^,  SOAT 2019^33^ |
| ChT average | $ 464 | $243 | $807 | SISMED^32^,  SOAT 2019^33^ |
| Adverse events | $ 1,521 | $1,178 | $2,231 | SISMED^32^,  SOAT 2019^33^ |
| 1st year remission | $ 109 | $76 | $150 | SISMED^32^,  SOAT 2019^33^ |
| ≥ 2nd year  remission | $ 53 | $32 | $83 | SISMED^32^,  SOAT 2019^33^ |
| DR regimen 1¶ | $4,518 | $4,184 | $4,861 | SISMED^32^,  SOAT 2019^33^ |
| DR regimen 2¶¶ | $4,657 | $4,312 | $5,010 | SISMED^32^,  SOAT 2019^33^ |
| DR average | $ 4,587 | $4,248 | $4,935 | SISMED^32^,  SOAT 2019^33^ |
| Palliative care | $ 248 | $213 | $300 | SISMED^32^,  SOAT 2019^33^ |
| ChT, chemotherapy; DR, distant recurrence  *AC-T: doxorubicin/cyclophosphamide x 4 cycles q/21days→Paclitaxel x 12  weeks  **TC: docetaxel/cyclophosphamide x 4 cycles q/21 days  ¶Palbociclib + Fulvestrant  ¶¶Ribociclib+ Fulvestrant | | | | |

Resources, costs and references used in the model.
